# Predictors of Plasma Levels of Direct Oral Anticoagulants Among Patients with Atrial Fibrillation in Need of Elective Cardiac Procedures

**DOI:** 10.1007/s10557-024-07573-1

**Published:** 2024-03-20

**Authors:** Vincenzo Russo, Eleonora Caiazza, Fiorella Chiara Delle Femine, Enrica Pezzullo, Sara Sarpa, Antonio Ianniciello, Caturano Alfredo, Antonello D’Andrea, Paolo Golino, Gerardo Nigro

**Affiliations:** 1 Cardiology Unit, Department of Medical Translational Sciences, University of Campania “Luigi Vanvitelli” – Monaldi Hospital, 80136 Naples, Italy; 2 Clinical Biochemistry Unit, Monaldi Hospital, Naples, Italy; 3 Department of Advanced Medical and Surgical Sciences, University of Campania Luigi Vanvitelli, Naples, Italy; 4 Cardiology Unit, Umberto I Hospital, Nocera Inferiore, Italy

**Keywords:** Direct oral anticoagulants, Atrial fibrillation, Plasma serum levels, Withdrawal period, Elective cardiac procedure, Bleeding risk

## Abstract

**Background:**

The withdrawal timing of direct oral anticoagulants (DOACs) among patients in need of elective invasive surgery is based on DOAC pharmacokinetics in order to perform the procedure out of the DOAC peak plasma concentration. We aimed to investigate the prevalence and predictors of plasma levels of DOACs out of trough range in patients with atrial fibrillation (AF) in need of elective cardiac procedure.

**Materials and Methods:**

We evaluated all consecutive AF patients on DOAC therapy in need of elective cardiac procedure, admitted to our division from January 2022 to March 2022. All patients underwent DOAC plasma dosing the morning of procedure day. They were categorized as in range, above range, and below range, according to the DOAC reference range at the downstream point. The timing of discontinuation of DOAC therapy was considered as appropriate or not, according to the current recommendations. The clinical predictors of out-of-range DOAC plasma levels have been evaluated.

**Results:**

We included 90 consecutive AF patients (56.6% male, mean age 72.95 ± 10.12 years); 74 patients (82.22%) showed DOAC concentration out of the expected reference range. In half of them (*n*, 37), the DOAC plasma concentration was below the trough reference range. Of the study population, 17.7% received inappropriate DOAC dosages (10% overdosing, 7% underdosing), and 35.5% had incorrect timing of DOAC withdrawal (26% prolonged, 9.5% shortened). At multivariable analysis, inappropriate longer DOAC withdrawal period (OR 10.13; *P* ≤ 0.0001) and increased creatinine clearance (OR 1.01; *P* = *0.0095*) were the independent predictors of plasma DOAC levels below the therapeutic trough range. In contrast, diabetes mellitus (OR 4.57; *P* = *0.001*) was the only independent predictor of DOAC plasma level above the therapeutic trough range.

**Conclusion:**

Increased creatinine clearance and inappropriate longer drug withdrawal period are the only independent predictors of DOAC plasma levels below the reference range; in contrast, diabetes is significantly correlated with DOAC plasma levels above the reference.

## Introduction

Direct oral anticoagulants (DOACs) are the first-line anticoagulant therapy for the treatment of atrial fibrillation (AF) in patients with increased thromboembolic risk [1]. The timing of DOACs discontinuation before elective invasive surgery is based on both surgery-related bleeding risk and the patient’s creatinine clearance [2]. Most of the cardiac invasive procedures are considered at minor bleeding risk. In these cases, DOACs should be discontinued at least 12 to 24 h before the procedure. This approach is based on DOACs pharmacokinetics in order to perform the procedure out of the DOACs peak plasma concentration. The aim of our exploratory study was to assess the plasma serum levels of DOACs among AF patients undergoing elective cardiac procedures and to investigate the predictors of out-of-range DOACs plasma levels.

## Materials and Methods

We included in the present study all consecutive AF patients taking any type and dosage of DOACs in need of elective cardiac procedure admitted to our division from January 2022 to March 2022. Patients who did not sign the informed consent (*n*, 4), with incomplete clinical (*n*, 1) and laboratory (*n*, 2) data were excluded from the analysis. Anthropometric, clinical, and laboratory data were collected; moreover, the pharmacologic therapy was reported. The timing of discontinuation of DOACs therapy was considered as appropriate or not, according to the current recommendations [2]. The morning of the procedure day, all patients underwent DOAC plasma dosing.

A specifically calibrated anti-factor Xa assay (Diagnostica Stago STArEvolution, Asnieres, France) was used to measure plasma concentrations of the factor Xa inhibitors (apixaban, rivaroxaban, and edoxaban). The diluted thrombin time test (HemosIL, Instrumentation Laboratory Belford, USA) was used for the quantitative assessment of dabigatran concentration. All patients were categorized as in range, above range, and below range according to the reference range at the downstream point specific for DOAC type and dosage (Table 1). The reference ranges used in our laboratory are based on the results of previous pharmacokinetic studies [3–6]. The clinical predictors of out-of-range DOAC plasma levels, both below and above, have been evaluated. The study was approved by the local ethics committee, and it was conducted in accordance to the Declaration of Helsinki and its later amendments.

**Table 1:** Reference range for DOAC peak and trough levels

	Reference range	
DOAC	Peak levels (ng/ml)	Trough levels (ng/ml)
Apixaban 5 mg	115–141	40–60
Apixaban 2.5 mg	39–85	17–25
Rivaroxaban 20 mg	184–343	12–137
Rivaroxaban 15 mg	178–313	18–136
Edoxaban 60 mg	388–444	268–336
Edoxaban 30 mg	376–412	130–174
Dabigatran 150 mg	74–383	28–155
Dabigatran 110 mg	52–275	40–215

*DOAC* direct oral anticoagulants

### Statistical Analysis

The distribution of continuous data was tested with Kolmogorov-Smirnov and Shapiro-Wilk tests. Normally distributed variables were expressed as mean ± standard deviation (SD). Categorical variables were reported as numbers and percentages. Continuous normally distributed variables were compared by using the Student *t*-test; differences between non-normally distributed variables were tested with the Mann-Whitney *U* test. Categorical variables were compared with chi-squared test, or Fisher exact test, when appropriate. Univariate and multivariate logistic regressions were used to investigate clinical variables independently associated with DOAC plasma levels below and above the through reference range. The variables included in the multivariate model were age, male gender, body mass index (BMI), creatinine clearance (CrCl), CrCl > 95 ml/min (below only), CrCl < 30 ml/min (above only), diabetes mellitus, inappropriate withdrawal period, and inappropriate DOAC dosage. We included variables with *P* < 0.05 by the univariable test as a candidate for the multivariable analysis, with a stepwise method. Multicollinearity was assessed using collinearity diagnostics. The variance inflation factors showed no significant collinearity (< 2.5) among the covariates. For all test, a *P* value < 0.05 was considered statistically significant. Analyses were performed by using R version 3.5.1 (R Foundation for Statistical Computing, Vienna, Austria).

## Results

**Fig. 1:**
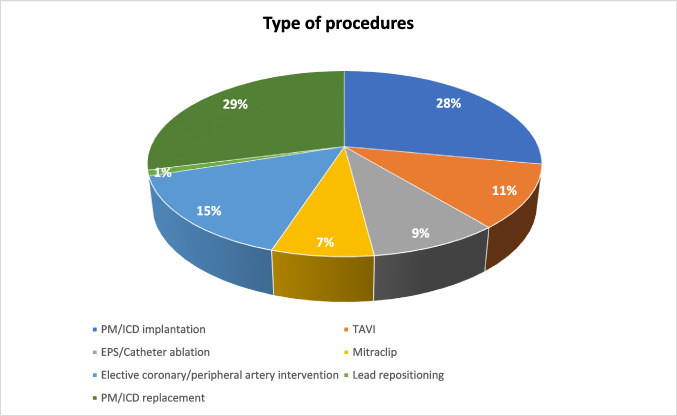
Elective cardiac procedures performed in study population

**Fig. 2:**
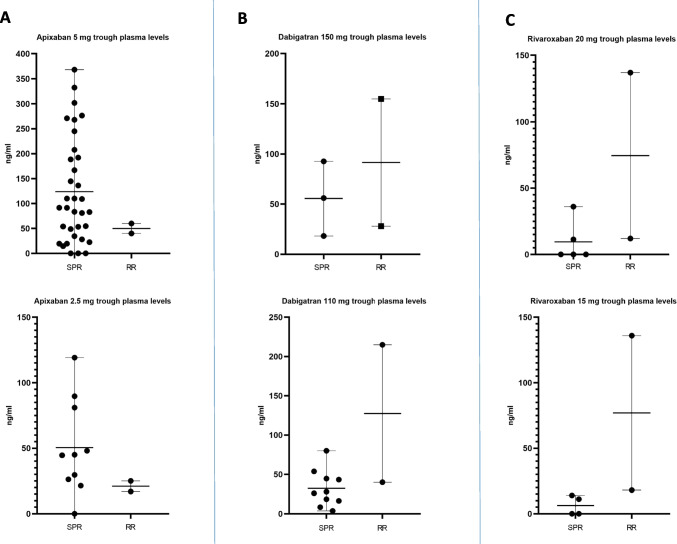
Scatterplot of individual study population data for different types and dosages of DOAC. *SPR* study population range; *RR* reference range

Ninety consecutive AF patients (56.6% male; 72.95 ± 10.12 years) were included in the study. The clinical characteristics of the study population are shown in Table 2. All patients underwent elective cardiac procedures as shown in Fig. 1. Of the study population, 17.7% was taking an inappropriate DOAC dosage (10% overdosing and 7% underdosing). In 35.5% of the study population, the timing of DOAC withdrawal was inappropriate (26% longer and 9.5% shorter). Seventy-four patients (82.22%) showed DOAC concentration out of the expected trough reference range; in half of them (*n*, 37), the DOAC plasma concentration was below the trough reference range. Figure 2 shows a scatter plot of individual data and reference range for different types and dosages of DOAC.

**Table 2:** Baseline characteristics of study population

	Overall population *n*, 90	In-range group *n*, 16	Out-of-range group *n*, 74	*P value*
Age, yrs	75 ± 10.12	77 ± 8.31	75 ± 10.47	0.48
Male sex (*n*, %)	51 (56.6)	8 (50)	43 (58.10)	0.55
BMI	27.73 ± 4.79	27 ± 3.5	28 ± 5	0.45
CHADS_2_-VA^2^Sc	4 ± 1.49	4 ± 1.4	4 ± 1.5	0.99
HASBLED	1 ± 0.88	1 ± 0.56	1 ± 0.93	0.99
Hypertension (*n*, %)	74 (82.22)	14 (87.5)	60 (81.08)	0.54
Diabetes (*n*, %)	37 (41.11)	3 (18.75)	34 (45.94)	0.04
Serum creatinine, mg/dl	1 ± 0.49	1.15 ± 0.4	1 ± 0.52	0.28
Creatinine clearance, ml/min	65 ± 31.67	68 ± 19.55	62.5 ± 33.62	0.53
CrCl > 95 ml/min (*n*, %)	17 (18.9)	0	17 (22.97)	0.034
CrCL< 30 ml/min (*n*, %)	6 (6.66)	0	6 (8.11)	0.24
Acetylsalicylic acid (*n*, %)	14 (15.55)	1 (6.25)	13 (17.56)	0.26
Clopidogrel (*n*, %)	7 (7.77)	2 (12.5)	5 (6.67)	0.43
Dabigatran (*n*, %)	13 (14.44)	7 (43.75)	6 (8.10)	0.0003
Rivaroxaban (*n*, %)	13 (14.44)	2 (12.5)	11 (14.86)	0.8
Apixaban (*n*, %)	55 (61.1)	7 (43.75)	48 (64.86)	0.11
Edoxaban (*n*, %)	9 (10)	0	9 (12.16)	0.13
Appropriate dosage (*n*, %)	74 (82.22)	14 (87.5)	60 (81.08)	0.54
Overdosed (*n*, %)	9 (10)	2 (12.5)	7 (9.45)	0.71
Underdosed (*n*, %)	7 (7.77)	0	7 (9.45)	0.2

*BMI* body mass index; *CHADS2VA2Sc* congestive heart failure, hypertension, age > 75 years, stroke, vascular disease, age 65–74 years, sex category; *HASBLED* hypertension, abnormal renal and liver functions, stroke, bleeding, labile international normalized ratio, elderly, drugs, or alcohol; *ClCr* creatinine clearance

At multivariable analysis, inappropriate longer DOAC withdrawal period (OR 1.15; *P* = *0.0001*) and clearance of creatinine > 95 ml/min (OR 8.04; *P* = *0.0041*) were independent predictors of plasma DOAC levels below the therapeutic trough range (Table 3). In contrast, diabetes mellitus (OR 5.07; *P* = *0.0008*) was the only independent predictor of DOAC plasma level above the therapeutic trough range (Table 4).

**Table 3:** Univariate and multivariate analyses for predictors of DOAC plasma level below the therapeutic trough range

	Univariate		Multivariate	
Variable	OR (95% CI)	*P*	OR (95% CI)	*P*
Age	0.1 (0.96–1.04)	0.94	−	−
Male gender	0.69 (0.29–1.61)	0.39	−	−
BMI	1.04 (0.95–1.14)	0.36	−	−
Hypertension	1.08 (0.34–3.32)	0.89	−	−
Diabetes	0.49 (0.18–1.06)	0.06	−	−
Creatinine clearance	1.02 (1.00–1.03)	0.0095	−	−
Creatinine clearance > 95 ml/min	7.09 (2.07–24.24)	0.0018	8.04 (1.93–3.44)	0.0041
Inappropriate excessive withdrawal period	10.13 (3.29–31.19)	0.0001	1.15 (1.07–1.22)	0.0001
Inappropriate low DOAC dosage	1.08 (0.23–5.14)	0.92	−	−

*BMI* body mass index; *DOAC* direct oral anticoagulants

**Table 4:** Univariate and multivariate analyses for predictors of DOAC plasma level above the therapeutic trough range

	Univariate		Multivariate	
Variable	OR (95% CI)	*P*	OR (95% CI)	*P*
Age	0.99 (0.94–1.03)	0.50	−	−
Male gender	1.77 (0.74–4.21)	0.19	−	−
BMI	0.99 (0.90–1.08)	0.85	−	−
Hypertension	0.56 (0.18–1.72)	0.31	−	−
Diabetes	4.57 (1.85–11.28)	0.001	5.07 (1.96–13.08)	0.0008
Creatinine clearance	0.98 (0.97–0.1)	0.04	0.98 (0.97–1.00)	0.07
Creatinine clearance < 30 ml/min	2.73 (0.47–15.79)	0.26	−	−
Inappropriate short withdrawal period	0.85 (0.19–3.79)	0.83	−	−
Inappropriate high DOAC dosage	1.52 (0.78–2.95)	0.21	−	−

*BMI* body mass index; *DOAC* direct oral anticoagulants

## Discussion

The main results of our study are the following: 82.2% of AF patients admitted to the hospital for elective cardiac procedure showed DOAC plasma levels out of the therapeutic trough range (41.1% below and 41.1% above). Creatinine clearance > 95 ml/min and the inappropriate longer drug withdrawal period are the only independent predictors of DOAC plasma levels below the reference range; in contrast, diabetes significantly correlated with DOAC plasma levels above the reference range.

According to the current recommendations, the correct timing of DOAC discontinuation before elective surgery depends on the patients’ clearance of creatinine, evaluated by Cockcroft-Gault formula, and on the hemorrhagic risk of the interventional procedure [2]. Many elective cardiac procedures are considered at minor hemorrhagic risk, carrying infrequent bleedings and with low clinical impact, and may be performed under minimally- or uninterrupted DOAC therapy (i.e., 12–24 h after last intake) [2]. This approach is based on DOAC pharmacokinetics and leads to perform the procedure at trough DOAC plasma level [7]. The management of DOACs may be challenging in several clinical scenarios or special populations and needs to be carefully evaluated [8]. Actually, few studies evaluated the DOAC plasma levels among AF patients in need of elective surgery [9].

In a sub-analysis of the Perioperative Anticoagulation Use for Surgery Evaluation (PAUSE) study, Shaw et al. [10] evaluated prespecified periprocedural-interruption strategy of DOACs among 2541 AF patients. A cut-off < 30 ng/mL, 30–49.9 ng/mL, and 50 ng/mL is empirically classified as undetectable or negligible, mildly elevated, and moderately elevated, respectively. Of the study patients, 79.4% showed preprocedural DOACs values considered safe (< 30 ng/mL). Among patients undergoing low-risk procedures, it was reported that age, female sex, use of a standard dose of DOACs, and a shorter DOAC discontinuation were associated with values > 30 ng/mL. Among patients in need of high-risk procedures, only weight and clearance were associated with DOAC levels > 30 ng/mL.

In a multicenter observational study including 422 patients who were taking DOACs, either for AF or venous thromboembolism (VTE), Godier et al. [11] aimed to determine the optimal duration of DOAC discontinuation that ensures a minimal anticoagulant effect at the day of invasive procedure (either elective or urgent). Applying the optimal DOAC discontinuation with a last DOAC intake 3 days before procedure, 77% of the study population achieved a minimal preprocedural DOAC plasma level ≤ 30 ng/mL. The duration of DOAC discontinuation, creatinine clearance < 50 mL/min, and antiarrhythmics were independent predictors of minimal preprocedural. Lastly, creatinine clearance < 50 mL/min, antiplatelets, and high-bleeding risk procedures were predictors of bleeding events.

Different from the previous studies [10, 11], we used, as reference ranges to classify the study population, the peak and trough serum levels specific for each type and dosage of DOACs. Moreover, we considered the discontinuation time suggested by the European Heart Rhythm Association (EHRA) recommendations to evaluate the withdrawal period as appropriate or inappropriate (longer or shorter).

Our results confirmed the relationship between creatinine clearance and DOAC plasma levels, since the increased creatinine clearance, in particular when it was higher than 95 ml/min, seems to be associated with DOAC plasma levels below the reference range. As expected, also the inappropriate longer drug withdrawal period was significantly correlated to it. Moreover, we firstly showed that diabetes may be associated with DOAC plasma levels above the reference range. Our speculative hypothesis is that this association may be mediated by the oxidative stress, a significant factor in the development and progression of diabetes [12], which may modulate the activity of both cytochrome (CYP) enzymes and glycoprotein (P-gp). In particular, the increased oxidative stress can inhibit the activity of CYP enzymes [13], leading to the altered metabolism and clearance of DOACs; moreover, it can modulate the activity of P-gp [14], affecting the efflux of DOACs from cells. The oxidative stress–induced modifications may potentially lead to the increased DOAC plasma levels among diabetic patients.

Finally, regarding the optimal DOAC withdrawal period before cardiac elective procedure, our results support the strategy suggested by EHRA recommendations based on the bleeding risk related to the procedure and the patient’s creatinine clearance. In case of diabetic patients, our results suggest that preprocedural DOAC plasma dosing might be part of a more careful patient-centered evaluation, especially in those with several risk factors for bleeding or undergoing procedures at high hemorrhagic risk.

### Limitations

Our study is limited by the relatively small number of patients; indeed, this is a single-center study reflecting the local practice and the generalization of results is limited. However, it is the only real-world observational study which included AF patients in need of elective cardiac procedures evaluated by DOAC plasma dosing and in whom the reference ranges are DOACs-specific and the withdrawal periods are not arbitrary. Moreover, we did not use high-performance liquid chromatography-mass spectrometry (HPLC/MS), currently considered the gold standard method for DOAC quantification; however, the assays used in our study provide drug measurements comparable to those measured with HPLC/MS [8].

## Conclusions

Among AF patients admitted to the hospital for elective cardiac procedure, 87.7% showed DOAC plasma levels out of the therapeutic trough range, equally distributed between above and below. Creatinine clearance > 95 ml/min and the inappropriate longer drug withdrawal period are the only independent predictors of DOAC plasma levels below the reference range; in contrast, diabetes significantly correlated with DOAC plasma levels above the reference.

## Data Availability

The data that support the findings of this study are available from the corresponding author, V.R., upon reasonable request.
